# “Walking in a maze”: community providers’ difficulties coordinating health care for homeless patients

**DOI:** 10.1186/s12913-016-1722-x

**Published:** 2016-09-07

**Authors:** Terri LaCoursiere Zucchero, Sarah McDannold, D. Keith McInnes

**Affiliations:** 1 Graduate School of Nursing, University of Massachusetts Medical School, 55 Lake Avenue North, Worcester, MA 01655 USA; 2 Center for Healthcare Organization and Implementation Research (CHOIR), Edith Nourse Rogers Memorial Veterans Hospital, 200 Springs Road, Bedford, MA 01730 USA; 3 Department of Health Law, Policy, and Management, Boston University School of Public Health, 715 Albany Street, Boston, MA 02118 USA

**Keywords:** Care coordination, Homelessness, Veterans

## Abstract

**Background:**

While dual usage of US Department of Veterans Affairs (VA) and non-VA health services increases access to care and choice for veterans, it is also associated with a number of negative consequences including increased morbidity and mortality. Veterans with multiple health conditions, such as the homeless, may be particularly susceptible to the adverse effects of dual use. Homeless veteran dual use is an understudied yet timely topic given the Patient Protection and Affordable Care Act and Veterans Choice Act of 2014, both of which may increase non-VA care for this population. The study purpose was to evaluate homeless veteran dual use of VA and non-VA health care by describing the experiences, perspectives, and recommendations of community providers who care for the population.

**Methods:**

Three semi-structured focus group interviews were conducted with medical, dental, and behavioral health providers at a large, urban Health Care for the Homeless (HCH) program. Qualitative content analysis procedures were used.

**Results:**

HCH providers experienced challenges coordinating care with VA medical centers for their veteran patients. Participants lacked knowledge about the VA health care system and were unable to help their patients navigate it. The HCH and VA medical centers lacked clear lines of communication. Providers could not access the VA medical records of their patients and felt this hampered the quality and efficiency of care veterans received.

**Conclusions:**

Substantial challenges exist in coordinating care for homeless veteran dual users. Our findings suggest recommendations related to education, communication, access to electronic medical records, and collaborative partnerships. Without dedicated effort to improve coordination, dual use is likely to exacerbate the fragmented care that is the norm for many homeless persons.

**Electronic supplementary material:**

The online version of this article (doi:10.1186/s12913-016-1722-x) contains supplementary material, which is available to authorized users.

## Background

Approximately 75 % of veterans who are enrolled in United States (US) Department of Veterans Affairs (VA) health care have another form of health coverage (e.g. Medicare, Medicaid, private health insurance) [1] and many obtain care from multiple health care systems each year. While this “dual use” may allow for increased health services access and choice, it is associated with negative consequences: poor communication among providers [2], incomplete or duplicate diagnosis and treatment plans [3, 4], fragmented services, diminished continuity and coordination of care, increased emergency department and hospital utilization, adverse events [5], and increased costs [6–8].

For the last few years, the VA has been contributing to the Obama administration’s initiative to end veteran homelessness. While housing is the priority concern, improving and preserving the health of homeless and formerly homeless veterans is also a goal. Veterans recently housed are at risk of losing their home due to physical or mental health deterioration.

The exact numbers of homeless veteran dual users are unknown. However, in 2014, federally funded Health Care for the Homeless (HCH) programs provided care to 21,504 veterans [9], equal to 45 % of the 47,725 US veterans identified as being homeless on a single night in January 2015 [10]. It is not known how many of these veterans are also receiving care at the VA, or where the remainder of homeless veterans not obtaining HCH services get their health care.

Veterans with multiple medical and psychosocial problems, such as the homeless, may be particularly susceptible to the negative consequences associated with dual use. For homeless individuals who tend to have unmet health needs [11], the opportunity to receive services from multiple organizations could be appealing. Yet little research has examined how community organizations serving homeless veterans interface and coordinate care with nearby VA medical centers. If care were well-coordinated, veterans may be receiving expert care from providers specialized in the needs of homeless persons (HCH providers) *and* the needs of veterans (VA providers). However, if care is disjointed, there is a risk that homeless veteran dual users may actually receive poor quality of care, leading to increased morbidity and mortality [6, 7]. The purpose of this study is to evaluate homeless veteran dual use by describing the experiences, perspectives, and recommendations of non-VA health professionals working in an urban HCH program, which serves a large number of veterans. We sought to identify coordination challenges that arise when vulnerable populations are potentially eligible for care in two different health care systems. While the focus is on a particular US population – military veterans who are homeless – the issues that are likely to arise are encountered in other countries where individuals and populations are in transitional states, for example moving from being housed to homeless, or from stable residence in their home country to refugee status in another country. State organizations exist to provide near universal health care, and yet non-governmental organizations also establish health care services and systems because of unique and/or acute needs of particular groups of people. Persons who move between these two systems create particular challenges for continuity of care, health care efficiency, and quality of care.

## Methods

### Study design

The interpretive nature of this study is grounded in the field of qualitative research.

Qualitative research approaches are an appropriate choice for the study of human experiences that are not well understood and/or when the voice of the group has been unexplored [12]; such is the case for non-VA health professionals working with homeless veterans in a HCH program. Focus group methodology is valuable when there is a belief that group interactions will lead to meaningful discourse that generates information and ideas unlikely to have arisen in one-on-one interviews [13]. In this study, where we were not only interested in gaps, challenges, and unmet needs, but also in potential solutions, the focus group format had advantages. We expected that, for example, a health professional might raise an issue that would encourage others to report related but different issues. Alternatively, when a participant raised a frustration or barrier, another participant might think of an innovative solution that he/she uses that might not have come out in an individual interview.

### Participants

After human subjects approval by the Institutional Review Board at the Bedford VA Medical Center, the study commenced in July 2014. The analysis of data ended in February 2015. Two hundred medical, dental, and behavioral health care providers at a large, urban Northeast HCH program serving approximately 1200 veterans per year were notified about the study. Eligible participants were required to have worked at the HCH for at least six months as a physician, registered nurse (RN) or nurse practitioner, physician assistant (a nationally certified and licensed health care professional who practices medicine under the supervision of a physician), dentist, psychologist, social worker, or case manager (social services support staff who advocates for and helps coordinate services for patients). Three broadcast recruitment emails were sent as follows – one month and one week prior to the start of the study and on the morning of each of the focus groups. We augmented this recruitment strategy by sending targeted emails to key informants through the assistance of the HCH’s Chief Operating Officer who had knowledge of staff experienced in working with veterans. Thirty health care providers from the HCH replied to the emails expressing interest in the study. However, the final number of participants in the focus groups totaled twenty (4, 6, and 10 participants respectively) as ten of the staff were not able to attend due to patient care responsibilities. The majority of participants were female (90 %). The sample included one physician, four RNs, six nurse practitioners, one physician assistant, one dentist, one social worker, and six case managers. Seven of the participants held administrative positions in addition to their clinical duties.

### Procedure

In November 2014, three focus groups were conducted at two downtown HCH program service sites. The groups occurred at the mid-day lunch break when providers and staff tend to have fewer patient-care duties. Focus groups lasted approximately 60 min, were audio-recorded, and eventually transcribed in their entirety. A semi-structured interview guide was developed with the input of three content experts in the separate areas of homeless health, veteran health, and care coordination. The final interview guide was reviewed by an experienced qualitative methodologist. The interview guide (see Additional file 1) sought to elicit HCH program provider experiences, perspectives, and recommendations regarding homeless veteran dual use. Each focus group was attended by two members of the research team. One of the senior investigators (TLZ or DKM) led the group, while another team member, a junior investigator (SM), took detailed field notes and ensured that no major topics were left unaddressed. This second member’s role was also to help elicit views from quieter participants. The moderator explained the format of the sessions and encouraged participants to freely express their opinions. Participants were asked to share their experiences communicating with VA, accessing patient information from the VA, and coordinating care for veterans. Participants were also asked to describe the advantages and disadvantages of veterans accessing care from both VA and HCH programs. Probing questions were used to further explore participant responses. Both senior investigators were experienced in focus group techniques and could gently pivot away from participants who seemed to be dominating the discussion, though this was a rare occurrence. Participants were given the opportunity to summarize their thoughts and feelings and to respond to the moderator’s impressions of the discussion’s main points. Before each session concluded, participants were asked if they had additional ideas that had not been discussed but were important in establishing better care for homeless veterans. Refreshments were provided during the focus groups and each participant received a gift card (USD 20) for participating in the study.

### Analysis

After each focus group, digital audio recordings were uploaded to a password-protected computer on a secure server and erased from the digital audio device. In the first phase of the analysis, one of the research team members (TLZ) became immersed in the data through transcribing the interviews verbatim, then reviewing the transcripts while listening to the audio file. This allowed her to reconnect to the focus group discussions and gain an overall understanding of the experiences of HCH providers. Margin notes were made to detail initial reflections. In the second phase, qualitative content analysis techniques [14] were used by this research team member (TLZ) who independently and systematically analyzed and hand-coded transcripts line-by-line. Provisional codes were assigned to organize the data into meaningful categories [15]. After hand-coding of the transcripts, the process was repeated by the same team member electronically. A code book was created [16] and all codes were then compiled using Microsoft Word into color-coded lists and relevant participant quotes were attached. During the third phase, all three transcripts were reviewed by another team member (SM), who examined the content of the codes. Agreement between the two investigators was high. For the few minor coding disagreements, discussion led to a consensus resolution [15]. Once the individual focus group data was analyzed, all coded data was combined into a data table and the entire research team (TLZ, SM, DKM) participated in the fourth and final phase of the analysis. Iterative procedures, as recommended by Hall et al. [17], were used to guide research team meetings in which coded data were reviewed and meaningful themes and exemplary quotes were identified and refined. Differences in interpretation were discussed until consensus was reached [15]. Throughout the study, an audit trail was maintained in order to increase trustworthiness [18] and included the following: study process, field, and reflexive notes; instrument development information; raw and coded data; and analytic decisions. In addition, credibility of the findings was assured through member checks [18] conducted by TLZ with five participants who verified that that their thoughts, feelings, and statements had been represented.

## Results

### Overview of findings

HCH program staff expressed frustration with the difficulty of establishing and maintaining communication with local VA medical centers. Four main themes emerged from the data --- insufficient knowledge of the VA and how to navigate its systems; lack of regular communication with VA medical centers and VA providers; desire for greater access to veterans’ VA medical information; and realization that the HCH program and VA have complementary expertise and resources. Participants perceived that these difficulties hampered efficient and effective care for homeless veterans. Themes are described in more detail below. Table 1 provides a summary of the themes with illustrative quotes and recommendations.

**Table 1:** Summary of 4 major themes, illustrative quotes, and recommendations for improving coordination

Theme	Illustrative quote	Recommendation
Frustrations Trying to Navigate the VA Health Care System “Black Box”	“Veterans don’t always know what they’re eligible for. It’s not printed out. You try to find out…I think if it’s hard for us, a veteran who might not be in the best of shape has to leap over hills and mountains, and it’s really tragic. I’m a veteran and I don’t even understand what people are eligible for.”	•“VA 101” training • In-person meetings between HCH and VA staff
Lack of Clear Lines of Communication with the VA	“Calling the VA, trying to get access to the medical providers to collaborate, or even just get appointments for the veteran is like calling a black hole. You get transferred and transferred and transferred and dropped. It is useless. I just stop taking the time and the effort to do it. I don’t have the time.”	• Clearly defined and transparent channels for phone and email communication • Transfer of patient-related information between institutions
Caring for Patients without Access to their VA Medical Record	“I think the biggest factor for me is lack of access to the medical record. Many, many times I am seeing people who want to establish primary care with us, but they’ve previously gone to the VA. Trying to get their health maintenance up to date with immunizations and colonoscopies etc. is so, so, difficult. I can easily place an order for a colonoscopy and get that scheduled, but they say, ‘Oh, I had one last year, but I’m not sure of the results.’”	• Access to VA electronic medical record • Use of a continuity of care record (CCR)
Encouraging Collaboration that Builds upon Complementary Expertise of the Two Organizations	“I think we’re ready to move in the direction of partnering with the VA….VA interest hasn’t been there but I think it could make a huge difference because then we can work together on these issues. This could mean better care -- better care for the individual but also for the overall system…. If the VA was willing to talk with the goal of increasing and strengthening a connection… it may mean that we each have to do things a little differently. That’s okay. We’ve always switched things up if it means better quality care for patients. Hopefully that’s how they would approach it too. But right now their lack of effort doesn’t make us feel like they’re taking care of their veterans.”	• Formal partnerships, such as between VA and the National Health Care for the Homeless Council

### Frustrations trying to navigate the VA health care system “Black Box”

A common sentiment among participants concerned their lack of understanding about the VA including the overall system, eligibility criteria to obtain VA care, and specific resources and services available to their homeless veteran patients. They expressed a need and desire for more knowledge to “navigate the VA” in order to provide better services to veterans.

*I mean we can read about the VA, but we don’t know what the VA does. We really don’t. So I think if they were willing to talk with folks, with the goal of increasing and strengthening the connection, and it was clear that this was the goal, it may mean we have to do things a little differently and they have to do things a little differently. Well then that’s okay. We’ve always switched things up if it means better quality care for patients. Hopefully that’s how the VA would approach it too.*

A case manager voiced frustration about trying to figure out how the VA works in order to get services for his patients and described the VA as a *“black box that can only be accessed by people within…”* Participants described how complicated and difficult it is to penetrate the VA system for outsiders such as themselves. Despite their efforts to learn more about the VA, it was difficult to provide guidance to their patients about how to access VA services.

*I’ve been a nurse here for over six years. The VA, it’s a very complicated system for me to navigate and I’ve had to help get patients connected, and there seems to be a lot of barriers. I’ve got a couple of patients who have come to me and said “I’m a vet and I’m starting to want to connect to the VA but I don’t really know how to.” I have to say, “I don’t really know either.”*

Participants suggested ways to improve VA and HCH program coordination, and increase HCH program staff knowledge of the VA. Recommendations included increased transparency of the VA, a VA website with both local and regional information important to homeless veterans and their non-VA care team, and a designated VA homeless “point person” who could serve as a resource and liaison for HCH and other community-based programs. Participants were very receptive to meetings or trainings about the VA system, which could lead to improved veterans’ health care.

*We need a ‘VA 101’ [training course]. We need to know what’s available, what does it mean, what’s an honorable discharge, what’s a dishonorable discharge, what are the criteria, what benefits are veterans eligible for, and here’s how you navigate the VA system.*

### Lack of clear lines of communication with the VA

Participants described making efforts to communicate with the VA in order to meet the needs of their homeless veteran patients. A common theme, however, was that there were no well-established and effective lines of communication with the VA. Some participants felt the situation negatively impacted their patients:

*I’ve had some really challenging situations when I’ve needed to contact the VA and get information so that I could better care for my patient. I have never had good results with being able to contact or communicate with anyone in the VA. Often I feel like I’m walking in a maze and the end result is that nobody can tell me who I need to talk to or how to get in touch with that person. So I feel like the care suffers, and the patient suffers because there’s a real disconnect with our ability to communicate directly with who we need to get in touch with.*

Several participants were concerned that communication problems jeopardized patient safety by increasing the likelihood of dangerous medication duplication and drug-drug interactions when homeless veterans receive prescriptions from both the VA and a non-VA organization. Medication reconciliation was recognized as an important activity between HCH program sites and the VA, but was usually not possible. Although the majority of the participants described challenges talking with the VA, two participants reported instances when communication went well, particularly during times of transition. One described the valuable communication with individual health care providers from a nearby VA outpatient clinic.

*Over the last couple of years, we’ve had a number of small encounters with the VA that have been really helpful for us. It’s always been where they’re about to discharge somebody and they just want to clarify that they can get the services that they want the patient to have when they get sent here. The VA knows the patient will be coming here, so they call our clinic and we’ve been able to have valuable conversations and been able to have a much smoother continuity of care for the patients. That’s been very, very, helpful.*

Despite inadequate communication with the VA currently, participants desired personal relationships with VA staff in order to improve communication processes in the future. Meeting each other face-to-face was seen as an important step toward good working relationships.

### Caring for patients without access to their VA medical record

Participants expressed concerns that not having access to the VA medical record was an “*insurmountable barrier”* to providing high quality care to their homeless veteran patients. Clinicians reported that because they did not have access to the medical records, tests and procedures often get duplicated.

*I think the biggest factor for me…is lack of access to the medical record. Many, many times I am seeing people who want to establish primary care with us, but they’ve previously gone to the VA. Trying to get their health maintenance up to date with colonoscopies and immunizations is so, so, difficult. I can easily place an order for a colonoscopy and get that scheduled, but they say, “Oh, I had one last year, but I’m not sure of the results.” Sometimes these patients are not always the best historians, so you’re kind of going blindly. It’s sometimes easier just to reorder basic labs, a chest x-ray, an EKG. If we had access to the VA [medical record] system, a lot of that could be avoided.*

For a clinician to access a VA medical record he or she must be credentialed by the VA in question. Despite considerable effort, VA credentialing has eluded providers.

*We’ve tried hard for years, but we have not been able to get our staff credentialed in the VA system. If you have a patient in front of you and you want to be able to see what is going on, wouldn’t it be great if we had staff who were VA credentialed. We have [credentialing] with other hospital systems but we haven’t been able to successfully be credentialed with [the VA].*

A few participants described that, in the absence of access to the VA medical record, the VA’s My Health*e*Vet online patient portal had been helpful in some cases. My Health*e*Vet had been used by some patients to share medical information such as labs and immunizations with their HCH providers. Unfortunately, assistance for veterans on how to use My Health*e*Vet was sporadic.

*The VA did a lot of work here in the shelter [affiliated with the HCH program] to help [veterans] get set up [on My HealtheVet]. They set up a computer downstairs. They sent a VA person here to help people get signed on. They spent a couple of months sending people here two or three days a week. They were really fabulous. We thought this is going to be great. Then they stopped sending people over here to help. Now if somebody wants to try to [use My HealtheVet], they have to figure it out on their own.*

### Encouraging collaboration that builds upon complementary expertise of the two organizations

Participants recognized that the HCH program and VA have complementary expertise. For example, the HCH has expertise in the medical and psychosocial health needs associated with homelessness, while the VA has expertise in veteran-related conditions and issues.

*At the end of their life [veterans] often have to [reconcile with] guilt and sorrow and remorse about what they have done in war… The VA knows to attend to those things. I think, as a Health Care for the Homeless organization, we could learn a lot from them. We need to learn how to send veterans off in a way that does not have their last breath being that they feel bad about what they did, what we asked them to do.*

Participants recognized that collaboration with the VA could help reduce duplication and costs. One noted the VA’s strong hepatitis C treatment program, indicating that without good communication the treatment process might also be initiated at the HCH program, causing duplicate services.

Participants discussed disappointment that the VA did not often reach out to them to collaborate despite the VA providers knowing that the veteran is receiving day-to-day care from the HCH. Nevertheless, participants had optimism that increased collaboration could be achieved, despite the current difficulties:

*I think it’s a universal frustration, a lack of being able to access medical records and communicate. It just feels outrageous in some ways, most of the time that people can’t get the care that they need. … If [the VA] can share their expertise with us and how to navigate the system… It would also be nice for us to share our expertise. So how do you become less rigid and more flexible?*

## Discussion

Dual use is a common phenomenon among veterans, and yet there is limited data about homeless veterans who are dual users, or about the non-VA organizations that care for these veterans in community settings. Despite the emphasis on US veterans, similar dual use and coordination issues are present in a variety of settings around the globe, for example in homeless health care in Europe [19] and refugee health programs in the Middle East and Europe [20].

In our study we found that health professionals caring for homeless veterans at a large HCH program experienced challenges coordinating care with VA medical centers for their patients who were dual users of the HCH and VA. The challenges included lack of knowledge about the VA health care system, insufficient communication between the HCH program and VA medical centers, and disappointment that HCH providers could not access the VA medical records of patients who received care from both the HCH program and the VA.

Our study provides important new insights into how non-VA health care providers from a large urban HCH program perceive the challenges and benefits of coordinating and collaborating with VA. It is the first study, to our knowledge, that explores through in-depth qualitative work the experience of a safety net provider organization trying to coordinate with a large national health care system for the care of homeless patients. Related findings have been documented elsewhere (though not specifically for a homeless veteran population), for example the inefficiencies and potential threats to quality of care from not having access to a patient’s medical record held by another system [21, 22]. Other findings, however, are distinct contributions of our study – such as the realization by HCH providers and staff of the unique cultural and medical knowledge that each organization brings: the HCH program has deep understanding of the medico-social needs of homeless persons, while the VA healthcare teams excel in knowledge of military culture and combat-related disorders relevant to veterans.

Although veteran dual users overall tend to be older, healthier, and have higher socio-economic status compared to veterans who exclusively use the VA for healthcare [23], sub-groups of dual users (such as our study population of homeless) may be at elevated risk for poor health. In VA mental health clinics, for example, dual using veterans have lower global functioning, are more likely to experience PTSD, obsessive-compulsive disorder, or substance abuse, and utilize more health care when compared to veterans using VA services only [24, 25].

Dual use creates discontinuities of care and inefficiencies (such as duplication of tests and procedures), leading to increased adverse events, morbidity, and mortality [2–8]. These problems may be elevated for homeless veterans who tend to have more complex medical (including oral health) and behavioral health problems, and who may lack social support. Further, mental illness and substance use, common among homeless persons, may hamper veterans’ ability to recall and reliably report to providers about healthcare services they received in another healthcare setting. Homelessness, and the lack of access to reliable internet, may reduce veterans’ ability to use the VA’s My Health*e*Vet patient portal which could otherwise be a means for veterans to share their VA medical information with non-VA providers [26].

Our findings are timely both in the US and in some other parts of the world, but for different reasons. In the US there have been calls from legislative, policy, and health leaders to make the VA more responsive to veterans’ desire for choice and convenience [27, 28]. Specifically, the VA’s 2014 strategic plan [27] identifies integration and coordination of VA and non-VA care as a priority goal. This attention is due to new legislation, such as the Veterans Access, Choice, and Accountability Act [28] and the Affordable Care Act [29], both of which are likely to increase opportunities for veterans to receive care from non-VA healthcare providers. This will bring greater focus on how care is coordinated between VA and non-VA providers.

In a different region of the globe, for example in European cites, care coordination issues have arisen in the care of highly vulnerable populations. Canavan et al. found in their study of 14 European capitals that there is a lack of coordination between non-governmental organizations who provide substantial amounts of services to homeless persons, and state providers of mental health services [19]. In the Middle East and Europe, dramatic flows of refugees have highlighted issues of coordination between emergency-focused medical organizations and the local country health care systems [20]. Not unlike homeless populations, refugees may have scattered medical records, and, due to linguistic and cultural barriers, and lack of trust, may be unable to adequately describe their own health histories.

### Implications

Our findings lead to recommendations in four areas: education, communication, access to electronic medical records, and collaborative partnerships. While these recommendations are focused on homeless veterans and arose in the context of HCH programs, they are likely adaptable to other kinds of dual use, for example non-homeless veterans receiving care in both a community health center and in a VA medical center, the homeless living anywhere, as well as refugees.

1. **Educate Staff from Both Organizations**

Participants suggested that through a “VA 101” course, the VA provide HCH programs with more information about their services and systems. Orientation materials about the military and military culture already exist [30], and could be adapted to give non-VA providers insights into the VA and the culture of veterans. Likewise, the VA could benefit from education about HCH programs. Joint VA and non-VA meetings and trainings (preferably in-person) about care coordination could be beneficial as well as provide opportunities to establish professional relationships and improve communication.

2. **Improve and Formalize Communication between VA and non-VA**

The Agency for Healthcare Research and Quality (AHRQ) identifies two types of communication necessary for coordinating care: *interpersonal communication* and *information transfer* [31]. Interpersonal communication, e.g. telephone conversations, emails, and face-to-face, would improve ties with the VA and produce more coordinated care of homeless veteran dual users. Participants suggested that each organization (HCH program and local VA) have a primary contact person to guide providers and staff to the services their organization offers and the channels for reaching those services. Health information transfer refers to exchange of data to communicate information about patients and their care [21, 31]. An important example of information transfer is the sharing of patient medical records described below.

3. **Establish Access to VA Electronic Medical Records**

Continuity across care settings would be enhanced by an accessible electronic medical record (EMR) [21]. Recent developments in the national government’s Office of the National Coordinator, and at the VA’s Office of Connected Care are encouraging the sharing of records across institutional boundaries. It is hoped that soon non-VA providers will have access to their veteran patients’ VA medical records. The VA, along with other health care entities, has already created an electronic continuity of care record (CCR) that is designed to be shared with non-VA providers [32]. This is a standardized, summary medical record that is designed to be electronically transmitted between organizations. Testing of the sharing of CCR could be implemented immediately with programs such as HCH programs.

4. **Collaborate through Formal Partnerships**

Formalized partnerships between VA and community providers are likely to improve care for homeless veteran dual users. The VA has recently established the Office of Community Engagement [33], a national resource that will serve as a “welcoming front door” (CM Clancy, MD, Interim Under Secretary for Health, Department of Veterans Affairs, All Employee Message, December 15, 2014) for outside organizations seeking to partner with the VA health care system. The VA should consider developing partnerships with national organizations that serve homeless veterans, such as the National Health Care for the Homeless Council, an umbrella organization supporting over 250 HCH programs across the country [34] which serve 21,000 homeless veterans annually [9]. Such a partnership would facilitate standardized care approaches and the spread of best practices between VA medical centers and HCH programs.

### Limitations

Our findings are limited because they come from one HCH organization, in one city. Additionally, they are based on only three focus groups with 20 health care providers, no patients, and no providers from the VA. As such, they may not be generalizable to HCH programs in other regions of the country. Our findings might have been different, for example, if we had interviewed health care providers in HCH programs in a smaller metropolitan area, or in a rural location. Nevertheless, our findings highlight important issues that have received little attention in prior literature and are especially relevant for highly vulnerable populations. Given the lack of information in this area, selecting a HCH program serving a large population of veterans offered a variety of perspectives from clinicians who have extensive experience with the issues around dual use.

## Conclusion

Our findings underscore existing challenges of coordinating care between two health systems, VA and non-VA, for a highly vulnerable population of homeless veterans. Without dedicated effort to improve coordination, dual use is likely to exacerbate the fragmented care that unfortunately is the norm for many homeless persons. The four recommendations provided above are consistent with the AHRQ’s Care Coordination Framework that includes teamwork, use of health information technology, sharing patient medical information, and helping with transitions of care [31]. While this study provides initial insights into provider perspectives on homeless veteran dual use, additional work is needed, for example to describe veteran perspectives on this kind of dual use. It will also be important to examine whether these findings are replicated in other settings that vary by socio-demographics, geography, and rurality, and in other health care organizations such as in community health centers which are not solely dedicated to serving homeless persons. Not surprisingly, disadvantaged populations tend to face greater barriers regardless of country or health care setting. An important goal of health policy should be to try to ensure all populations have access to and receive high quality care. More emphasis on care coordination will contribute to this goal.

## Additional file

Additional file 1: Semi-structured focus group interview guide. (DOC 105 kb)

## Abbreviations

AHRQ: Agency for Healthcare Research and Quality
CCR: Continuity of care record
HCH: Health care for the Homeless
RN: Registered nurse
PTSD: Post-traumatic stress disorder
VA: Department of Veterans Affairs
