# Supplementary material for: “Walking in a maze”: community providers’ difficulties coordinating health care for homeless patients
Source: BMC Health Serv Res. 2016 Sep 7;16(1):480. doi: 10.1186/s12913-016-1722-x (PMC5015348; doi:10.1186/s12913-016-1722-x)
Supplement: Additional file 1: — Semi-structured focus group interview guide. (DOC 105 kb) [file 12913_2016_1722_MOESM1_ESM.doc]

**Semi-structured Focus Group Interview Guide**

| Overview of topics covered:  A. EXPERIENCES with homeless Veteran dual users  B. COORDINATION OF CARE  C. SUGGESTIONS TO IMPROVE COORDINATION OF CARE |
| --- |

**A. EXPERIENCES with homeless Veteran dual users**

Let’s start by talking about any experiences you’ve had working with homeless Veterans that were “dual users” (Vets that received care/services at [the Health Care for the Homeless (HCH) program program] and at the VA).

First, we’d like to hear about **positive experiences** with homeless Veteran dual use.

- Can anyone share a positive experience they’ve had?

Prompts:

*What made this a positive experience?*

*How did the Veteran benefit or not benefit from this experience?*

Now we’d like to hear about any **negative experiences** with homeless Veteran dual use.

- Can anyone share a negative experience they’ve had?

Prompts:

*What made this a negative experience?*

*How did the Veteran benefit or not benefit from this experience?*

*What was the most challenging aspect of this experience?
What would have helped this experience?*

**B. COORDINATION OF CARE between [the HCH program] and VA, as well as the Veteran**

# Now I’d like to talk about coordination of care for homeless Veterans who are dual users and receive care (or could receive care) at the VA and outside of the VA.

# Just to be clear, by “coordination of care” we mean the planned integration of patient care activities between two or more participants or organizations involved in a patient’s care to facilitate or aid in the appropriate delivery of health care services.

- Can you describe the barriers to care coordination between [the HCH program] and the VA?

Prompts:

*Why is this a barrier or problem for coordination of care?*

- Can you describe the facilitators to care coordination between [the HCH program] and the VA?

Prompts:

*Why is this a facilitator or why does this help with coordination of care?*

Now let’s talk about the patient’s involvement in care coordination. Again, for this study we are interested in homeless Veterans who use care/services at the VA and outside of the VA, such as the HCH program.

- To begin with, what are your thoughts about homeless Veterans being involved in care coordination?

Let’s think specifically about barriers.

- Can you describe the barriers to homeless Veteran’s being involved in care coordination between [the HCH program] and the VA?

And now let’s talk about facilitators or things that help with care coordination.

- Can you describe the facilitators (or what would help) with homeless Veteran’s being involved in care coordination between [the HCH program] and the VA?

**C. SUGGESTIONS TO IMPROVE COORDINATION OF CARE between [the HCH program] and VA**

We’re interested in hearing about your ideas/suggestions to improve coordination of care between [the HCH program] (and other non-VA organizations) and the VA so that homeless Veterans can receive quality care and better outcomes.

Let’s start by talking about organizational strategies and then we’ll discuss individual staff strategies.

- What are some organizational strategies that [the HCH program] could do to improve coordination of care between [the HCH program] and the VA?

Prompts:

*How would this work?*

*Who would be responsible?*

- What are some organizational strategies that the VA could do?

Now let’s discuss individual strategies to improve care coordination between [the HCH program] and the VA.

- What are some individual strategies that [the HCH program] staff could do to improve coordination of care between [the HCH program] and the VA?
- What are some individual strategies that VA staff could do?

# CONCLUSION

Let’s begin to conclude the discussion. We’ve learned a lot today about your experiences and thoughts regarding dual use by homeless Veterans.

We learned --- *RECAP MAJOR FINDINGS/THEMES* (Experiences, Care Coordination, Suggestions for Improved Care Coordination)

- In the last couple minutes before ending, is there anything else that you would like to briefly add about the topic of dual use by homeless Veterans?

Thank you all very much for your very valuable participation in today’s focus group.
